# Ultrasound radiomics-based machine learning models for risk stratification of follicular thyroid tumors

**DOI:** 10.3389/fonc.2025.1707586

**Published:** 2026-01-05

**Authors:** Ya Yuan, Xinyue Wang, Hongyan Deng, Kunpeng Cao, Fei Yu

**Affiliations:** 1 Department of ultrasound, The First Affiliated Hospital With Nanjing Medical University, Nanjing, China; 2 Department of nuclear medicine, The Shanghai Tenth Clinical Medical College with Nanjing Medical University, Shanghai, China

**Keywords:** follicular thyroid tumors, machine learning, peritumoral radiomics, risk stratification, ultrasound

## Abstract

**Background:**

Follicular thyroid carcinoma (FTC) is the second most common malignant thyroid tumor. Preoperative differentiation among follicular thyroid adenoma (FA), follicular tumor of uncertain malignant potential (FT-UMP), and FTC remains challenging using conventional ultrasound and fine-needle aspiration. This study aims to develop a machine learning model utilizing ultrasound radiomic features to improve risk stratification of follicular thyroid tumors.

**Methods:**

A total of 277 patients with histopathologically confirmed follicular tumors (163 FA, 63 FT-UMP, 51 FTC) were included. Clinical and ultrasound features, along with radiomic features from intratumoral and peritumoral regions, were extracted from preoperative ultrasound images. Three machine learning models—logistic regression (LR), support vector machine (SVM), and random forest (RF)—were trained to construct four models: clinical-ultrasound (U), clinical-ultrasound with intratumoral radiomics (UI), clinical-ultrasound with peritumoral radiomics (UP), and clinical-ultrasound with combined intratumoral and peritumoral radiomics (UIP).

**Results:**

The RF-based clinical-ultrasound model demonstrated the highest accuracy (test: 0.643) but exhibited significant overfitting in radiomics-based models. The SVM model showed moderate performance. The LR model in the UP and UIP models delivered stable performance, achieving the highest test accuracy of 0.643. Specifically, the UP model showed improved micro-AUC, specificity, negative predictive value (NPV), and F1 score. The LR model exhibited high sensitivity but low specificity for benign nodules, and high specificity but low sensitivity for malignant nodules. All models performed poorly in identifying FT-UMP nodules.

**Conclusion:**

Integrating peritumoral radiomic features with clinical-ultrasound features using logistic regression enhances the differentiation between benign and malignant follicular thyroid tumors.

## 1 Introduction

Follicular thyroid carcinoma (FTC) is the second most common thyroid malignant tumor, accounting for about 17% of all thyroid cancers (1). About 10–15% of FTC patients develop distant metastases to the lungs and bones, leading to poorer survival outcomes compared to papillary thyroid carcinoma (PTC) (2–4). Thus, early diagnosis of FTC is crucial for treatment and prognosis.

Ultrasound is the primary imaging modality for evaluating thyroid nodules, enabling classification based sonographic features and assessment of malignant potential (5). The currently widely used TI-RADS classification systems are built on this, while they are mainly established based on the ultrasound features for PTC, and have certain limitations in the diagnosis of follicular tumors (6, 7). In ultrasonographic images, the FTC has some similarities with benign (Follicular Thyroid Adenoma,FA) and low-risk (Follicular Tumor of Uncertain Malignant Potential, FT-UMP) nodules (8). Even fine-needle aspiration (FNA) cannot reliably differentiate among these entities (9). Only complete removal of the lesion and exploration of its capsular infiltration can clearly determine the specific type of the nodules (10, 11). In addition, the clinical management of low-risk nodules is also controversial.2025 ATA Management Guidelines for Adult Patients with Differentiated Thyroid Cancer state that further treatment with completion thyroidectomy/lymphadenectomy and/or radioactive iodine therapy for low-risk nodules like FT-UMP is not advised routinely. The optimal approach to monitoring of these tumors should be determined according to the surgical approaches, laboratory findings, and the patient’s wishes (12). Therefore, extracting more diagnostic information from ultrasound images to find a more accurate method for differentiating follicular tumors, so as to reduce unnecessary surgeries/therapies, is currently the focus of research. Previous studies (13–15) have mostly focused on differentiating FTA and FTC, with limited attention to low-risk follicular tumors. It is worth noting that FT-UMPs are defined as well-differentiated thyroid tumors with follicular architecture that are encapsulated or unencapsulated but well-circumscribed, in which invasion remains questionable after thorough sampling and exhaustive examination (16, 17). That means FT-UMP is generally an indolent disease, but some patients still show distant recurrence. Due to its inherent uncertainties, there are certain challenges in determining the clinical follow-up period or making surgical decisions (18).

The aim of this study is to develop a hierarchical model based on ultrasound images using machine learning methods, in order to assess the risk stratification of follicular tumors and provide a reference for clinical decision-making.

## 2 Method

### 2.1 Patients

From January 2020 to September 2023, 277 patients with histopathologically confirmed follicular thyroid tumors (163 FTA, 63 FT-UMP, 51 FTC) were included. Preoperative neck ultrasound was performed by an operator with over 10 years of experience using EPIQ5 (Philips Healthcare) equipped with a 6–18 MHz linear transducer. The following sonographic features were recorded: nodule number (single, multiple); location (upper, middle, lower, isthmus); maximum diameter (≤1 cm, 1–4 cm, >4 cm); echogenicity (hyperechoic, isoechoic, hypoechoic); aspect ratio (<1, ≥1); composition (solid, predominantly solid, predominantly cystic); calcifications (absent,microcalcification,macrocalcification,rim calcification);shape (round-to-oval, irregular); margin (smooth, spiculated, unclear); halo (absent, regular complete, irregular interrupted); blood flow (grade 0: no signal; grade 1: partial clear signals; grade 2: moderate signals; grade 3: abundant signals); elasticity score (1–5).Ultrasound features were independently evaluated by 2 attending physicians with more than 5 years of experience in the thyroid imaging. They had no prior knowledge of the pathological findings. Features in which there was disagreement were recorded after agreement between the two physicians. Features with intraclass correlation coefficients (ICCs) greater than the reproducibility threshold of 0.9, both in intraobserver and interobserver assessments, were screened out for subsequent analysis.

### 2.2 Ultrasound radiomic analysis

#### 2.2.1 Clinical and ultrasound features

Univariate and multivariate logistic analysis(ordered multinomial logistic regression) was conducted on clinical and ultrasound features. The response variable is a ordinal variable with three classes. Features with a statistical value of P < 0.05 were included in the subsequent analysis.

#### 2.2.2 Radiomic features

ITK-SNAP 3.6.0 was used to manually delineate intratumoral regions of interest (ROIs) on DICOM images of the maximum cross section on grayscale US images, which was regarded as the intratumoral ROI. Peritumoral ROIs were generated by expanding the intratumoral boundary outward by 2 voxels. To appraise the feature reliability, reader 1 and reader 2 (with 5 years of experience in thyroid imaging) independently outlined ROIs on images from 30 randomly selected patients. Reader 2 repeated the same process within 1 week. The intra and interobserver reproducibility were assessed by the ICC, and an ICC greater than 0.9 indicated good consistency. Radiomics features were automatically extracted using an open-source Python package, PyRadiomics (https://pyradiomics.readthedocs.io/).

Feature values were normalized via Z-score transformation (Equation 3) (19). The normalization method is as follows:


$$column\;=\frac{column\;-\;mean}{std}$$

column: represents a certain column (feature) in the dataset; mean: the mean of this column; std: the standard deviation of this column.

One-way ANOVA was conducted on all the normalized ultrasound radiomics features of FTA, FT-UMP and FTC groups, and only those with P < 0.05 were retained. The Pearson correlation coefficient was used to calculate the correlation between features. To maximize the discriminative power of radiomics features, we employed a correlation-based greedy recursive backward elimination strategy for feature selection. This method iteratively identifies and removes the most redundant features based on internal correlation structure. In each iteration, we computed absolute pairwise correlation coefficients among all remaining features and calculated the average correlation for each feature. The feature showing the highest average correlation, indicating the least unique information was eliminated. This process repeated recursively until the maximum pairwise correlation among remaining features fell below a predefined threshold(0.9). As a model-agnostic preprocessing approach, this method effectively mitigates multicollinearity while preserving the most representative features, thereby enhancing model robustness and interpretability. By explicitly addressing feature redundancy, our approach provides a computationally efficient framework for feature subset selection.

The selected features was randomly divided into a training cohort and a test cohort at a ratio of 8:2. Then, we input the features into a polynomial logistic regression model with L1 penalty term of the Least Absolute Shrinkage and Selection Operator (LASSO) algorithm to directly solve the three-classification problem. The penalty coefficient λ of LASSO was optimized for selection by 5-fold cross validation. The final selected results were included in the subsequent analysis.

### 2.3 Model construction

These features (clinical and ultrasound and radiomic features) were then input into machine learning models (Logistic Regression, LR;Support Vector Machine, SVM;Random Forest, RF) to construct intratumoral and intratumoral-peritumoral dual-region radiomics ultrasound radiomics models. Stratified five-fold cross-validation was used to find the optimal parameters of the models and determine the final models.

We also included a SHapley Additive exPlanations(SHAP) (20)method to enhance the transparency and explainability of the best-performing model by prioritizing the importance of features, in terms of assessing the contribution to model performance.

**Figure 1:**
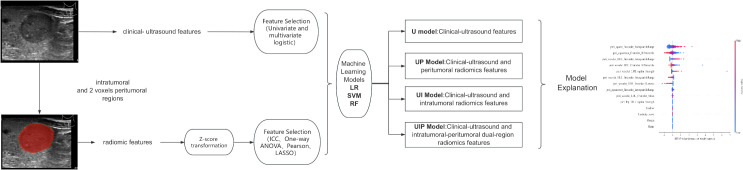
Workflow of research. .

The workflow of this study is presented in **Figure 1**.

This study was approved by the Ethics Committee on October 25.2023.with the approval no.2023-SR-691.This study complied with the Declaration of Helsinki.

### 2.4 Statistical analysis

Statistical analyses were performed using IBM SPSS 26.0 (IBM, New York, USA). The normality of the data was verified via the Kolmogorov-Smirnov test. Continuous data conforming to a normal distribution are expressed as the mean ± standard deviation (SD) and were compared using one-way ANOVA; non-normal data are expressed as the median [interquartile range (IQR)] and were compared using the Kruskal-Wallis H test. Categorical data are expressed as numbers (%) and were compared using a chi-square test or Fisher’s exact test, as appropriate. A P < 0.05 represented a statistically significant difference.

## 3 Result

**Figure 2:**
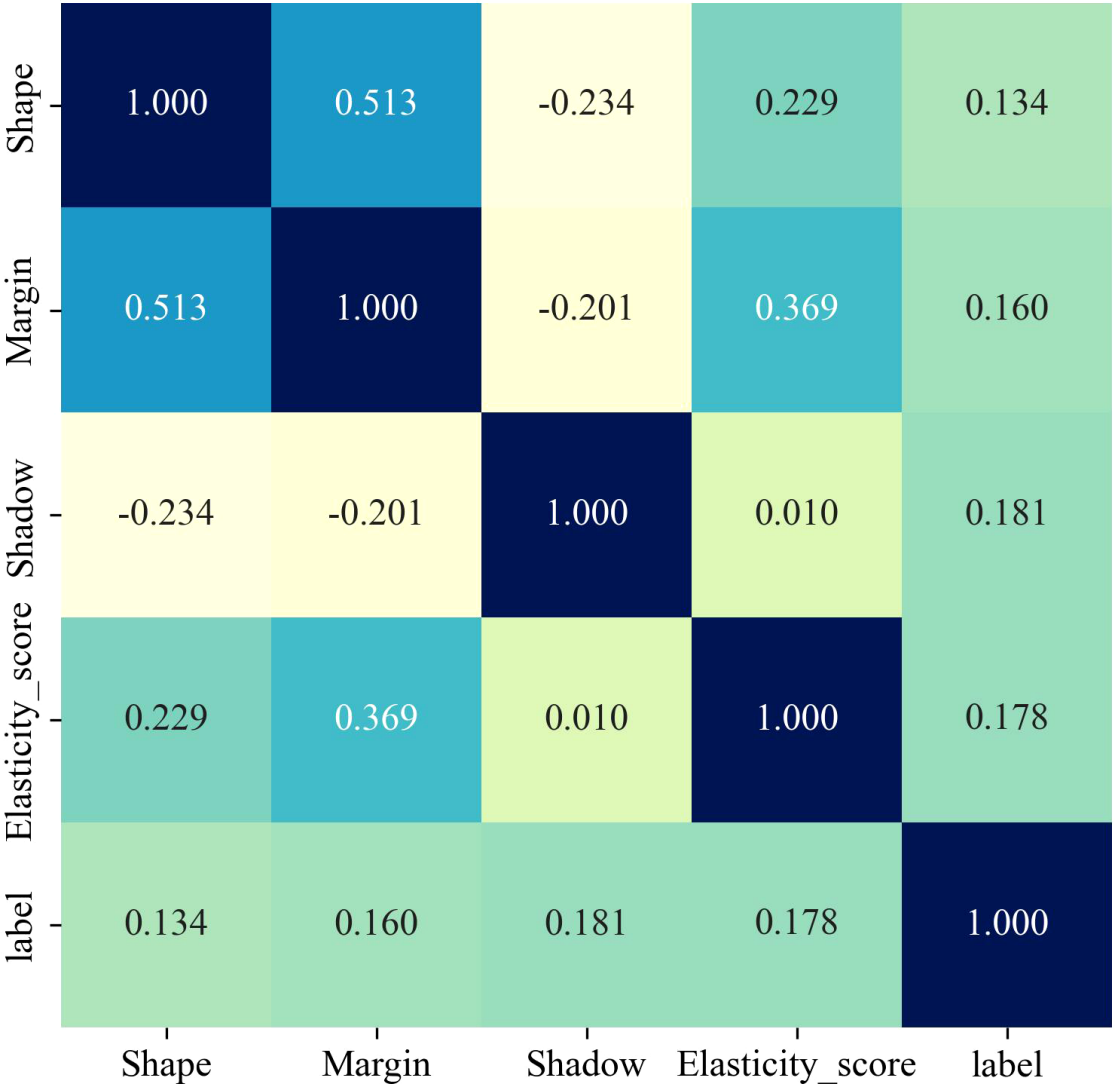
Spearman correlation of each clinical and ultrasound feature.

The clinical and ultrasound features in the training cohort and the test cohort (shown in **Table 1**) were included in the univariate and multivariate logistic regression analysis. The results (**Table 2**, **Figure 2**) showed that shape, margin, shadow and elasticity score(P<0.05) were independent risk factors. Based on these factors, machine learning models were constructed.

**Table 1:** The clinical and ultrasound features in the training cohort and the test cohort.

Features	Training cohort				Test cohort			
	Benign	Low-risk	Malignant	P value	Benign	Low-risk	Malignant	P value
Age M (Q_1_, Q_3_)	45.000 (34.000, 56.000)	51.000 (39.000, 59.250)	42.000 (28.000, 57.000)	0.096	48.000 (40.000, 57.000)	52.000 (39.000, 55.000)	36.000 (31.250, 46.250)	0.149
Gender				0.265				0.651
male	39(30.000)	9(18.000)	11(26.829)		9(27.273)	3(23.077)	4(40.000)	
female	91(70.000)	41(82.000)	30(73.171)		24(72.727)	10(76.923)	6(60.000)	
Nodule Number				0.227				0.519
single	110(84.615)	46(92.000)	38(92.683)		30(90.909)	13(100.000)	9(90.000)	
multiple	20(15.385)	4(8.000)	3(7.317)		3(9.091)	–	1(10.000)	
Location				0.148				0.437
upper	19(14.615)	9(18.000)	5(12.195)		7(21.212)	1(7.692)	2(20.000)	
middle	39(30.000)	23(46.000)	19(46.341)		14(42.424)	10(76.923)	5(50.000)	
lower	65(50.000)	17(34.000)	17(41.463)		9(27.273)	2(15.385)	3(30.000)	
iethmus	7(5.385)	1(2.000)	–		3(9.091)	–	–	
Nodule max diameter				0.005				0.626
≤1 cm	9(6.923)	5(10.000)	–		3(9.091)	–	–	
1-4cm	74(56.923)	31(62.000)	15(36.585)		18(54.545)	8(61.538)	5(50.000)	
>4 cm	47(36.154)	14(28.000)	26(63.415)		12(36.364)	5(38.462)	5(50.000)	
Echogenicity				0.007				0.267
hypoechoic	21(16.154)	12(24.000)	18(43.902)		8(24.242)	5(38.462)	5(50.000)	
isoechoic	107(82.308)	37(74.000)	22(53.659)		25(75.758)	8(61.538)	5(50.000)	
hyperechoic	2(1.538)	1(2.000)	1(2.439)		–	–	–	
Aspect Ratio				0.173				1
<1	129(99.231)	48(96.000)	41(100.000)		33(100.000)	13(100.000)	10(100.000)	
≥1	1(0.769)	2(4.000)	–		–	–	–	
Composition				0.037				0.194
solid	53(40.769)	25(50.000)	27(65.854)		16(48.485)	5(38.462)	8(80.000)	
predominantly solid	65(50.000)	20(40.000)	14(34.146)		12(36.364)	4(30.769)	2(20.000)	
predominantly cystic	12(9.231)	5(10.000)	–		5(15.152)	4(30.769)	–	
Calcifications				<0.001				0.089
absent	91(70.000)	35(70.000)	14(34.146)		25(75.758)	9(69.231)	6(60.000)	
rim calcification	8(6.154)	2(4.000)	2(4.878)		2(6.061)	–	–	
microcalcification	25(19.231)	5(10.000)	7(17.073)		4(12.121)	–	–	
macrocalcification	6(4.615)	8(16.000)	18(43.902)		2(6.061)	4(30.769)	4(40.000)	
Shape				0.052				0.097
round to oval	121(93.077)	46(92.000)	33(80.488)		29(87.879)	13(100.000)	7(70.000)	
irregular	9(6.923)	4(8.000)	8(19.512)		4(12.121)	–	3(30.000)	
Margin				<0.001				0.154
smooth	124(95.385)	47(94.000)	34(82.927)		30(90.909)	13(100.000)	8(80.000)	
spiculated	5(3.846)	3(6.000)	–		2(6.061)	–	–	
unclear	1(0.769)	–	7(17.073)		1(3.030)	–	2(20.000)	
Shadow				0.002				0.464
absent	93(71.538)	28(56.000)	23(56.098)		20(60.606)	7(53.846)	5(50.000)	
regular complete halo	33(25.385)	19(38.000)	10(24.390)		12(36.364)	4(30.769)	3(30.000)	
irregular interrupted halo	4(3.077)	3(6.000)	8(19.512)		1(3.030)	2(15.385)	2(20.000)	
Blood Flow				0.028				0.670
0	8(6.154)	1(2.000)	–		3(9.091)	–	–	
1	6(4.615)	3(6.000)	–		–	–	–	
2	26(20.000)	12(24.000)	2(4.878)		4(12.121)	2(15.385)	1(10.000)	
3	90(69.231)	34(68.000)	39(95.122)		26(78.788)	11(84.615)	9(90.000)	
Elasticity score				0.011				0.083
1	1(0.769)	–	–		–	–	–	
2	75(57.692)	28(56.000)	12(29.268)		19(57.576)	8(61.538)	2(20.000)	
3	51(39.231)	19(38.000)	29(70.732)		14(42.424)	5(38.462)	8(80.000)	
4	3(2.308)	3(6.000)	–		–	–	–	
5	–	–	–					

Values are presented as count (%).

Differences were considered statistically significant at P<0.05.

**Table 2:** Univariate and multivariate logistic regression analysis results of clinical and ultrasound features.

Variables	Univariate					Multivariate				
	β	S.E	t	*P*	OR (95%CI)	β	S.E	t	*P*	OR (95%CI)
Gender										
male					1.00 (Reference)					
female	0.2	0.27	0.72	0.473	1.22 (0.71 ~ 2.08)					
Nodule Number										
single					1.00 (Reference)					
multiple	-0.73	0.43	-1.72	0.086	0.48 (0.21 ~ 1.11)					
Location										
upper					1.00 (Reference)					
middle	0.45	0.35	1.28	0.2	1.57 (0.79 ~ 3.11)					
lower	-0.15	0.36	-0.42	0.673	0.86 (0.42 ~ 1.74)					
iethmus	-1.9	1.09	-1.74	0.081	0.15 (0.02 ~ 1.27)					
Nodule max diameter										
≤1 cm					1.00 (Reference)					
1-4cm	0.53	0.54	0.97	0.331	1.69 (0.59 ~ 4.88)					
>4 cm	0.99	0.55	1.8	0.072	2.69 (0.92 ~ 7.88)					
Echogenicity										
hypoechoic					1.00 (Reference)					1.00 (Reference)
isoechoic	-1.02	0.27	-3.78	**<.001**	0.36 (0.21 ~ 0.61)	-1.38	0.34	-4.06	**<.001**	0.25 (0.13 ~ 0.49)
hyperechoic	-0.37	0.97	-0.38	0.706	0.69 (0.10 ~ 4.67)	-0.85	1.02	-0.83	0.404	0.43 (0.06 ~ 3.16)
Aspect Ratio										
<1					1.00 (Reference)					
≥1	0.37	0.95	0.39	0.694	1.45 (0.22 ~ 9.39)					
Composition										
solid					1.00 (Reference)					1.00 (Reference)
predominantly solid	-0.66	0.25	-2.6	**0.009**	0.52 (0.31 ~ 0.85)	-0.21	0.31	-0.67	0.505	0.81 (0.44 ~ 1.49)
predominantly cystic	-0.83	0.43	-1.93	0.053	0.44 (0.19 ~ 1.01)	-1.14	0.58	-1.98	**0.048**	0.32 (0.10 ~ 0.99)
Calcifications										
absent					1.00 (Reference)					1.00 (Reference)
rim calcification	-0.23	0.61	-0.38	0.704	0.79 (0.24 ~ 2.61)	0.69	0.7	0.98	0.326	2.00 (0.50 ~ 7.94)
microcalcification	-0.16	0.38	-0.42	0.671	0.85 (0.41 ~ 1.78)	-0.77	0.51	-1.52	0.127	0.46 (0.17 ~ 1.25)
macrocalcification	2.04	0.34	5.96	**<.001**	7.68 (3.93 ~ 15.01)	2.78	0.45	6.17	**<.001**	16.07 (6.65 ~ 38.81)
Shape										
round to oval					1.00 (Reference)					1.00 (Reference)
irregular	0.84	0.39	2.14	**0.032**	2.31 (1.07 ~ 4.96)	-1.81	0.71	-2.54	**0.011**	0.16 (0.04 ~ 0.66)
Margin										
smooth					1.00 (Reference)					1.00 (Reference)
spiculated	-0.58	0.68	-0.85	0.393	0.56 (0.15 ~ 2.12)	-15.83	0.49	-32.51	**<.001**	0.00 (0.00 ~ 0.00)
unclear	2.97	0.8	3.69	**<.001**	19.51 (4.03 ~ 94.47)	5.63	1.15	4.91	**<.001**	279.02 (29.48 ~ 2640.70)
Shadow										
absent					1.00 (Reference)					1.00 (Reference)
regular complete halo	0.28	0.26	1.08	0.281	1.33 (0.79 ~ 2.22)	0.81	0.34	2.41	**0.016**	2.26 (1.16 ~ 4.37)
irregular interrupted halo	1.72	0.46	3.78	**<.001**	5.60 (2.29 ~ 13.69)	2.12	0.63	3.35	**<.001**	8.36 (2.42 ~ 28.97)
Blood Flow										
0					1.00 (Reference)					1.00 (Reference)
1	1.55	1.24	1.25	0.211	4.73 (0.41 ~ 54.14)	-0.68	1.49	-0.46	0.647	0.51 (0.03 ~ 9.30)
2	1.74	1.08	1.6	0.109	5.68 (0.68 ~ 47.38)	1.57	1.12	1.4	0.162	4.80 (0.53 ~ 43.30)
3	2.25	1.05	2.14	**0.032**	9.53 (1.21 ~ 74.86)	1.26	1.08	1.16	0.246	3.52 (0.42 ~ 29.43)
elasticity score										
1					1.00 (Reference)					1.00 (Reference)
2	13.95	0.23	61.69	**<.001**	1139545.58 (731645.99 ~ 1774853.06)	18.26	0.4	45.42	**<.001**	85332689.84 (38803681.67 ~ 187654048.30)
3	14.7	0.23	64.47	**<.001**	2424724.69 (1550837.14 ~ 3791042.71)	18.42	0.42	44.35	**<.001**	100165968.84 (44376073.94 ~ 226095290.18)
4	14.28	0.55	26.03	**<.001**	1592077.02 (543234.89 ~ 4665954.38)	36.16	0.49	74.28	**<.001**	5036124771593869.00 (1939834166018742.75 ~ 13074598416376352.00)
Age	0	0.01	0.47	0.641	1.00 (0.99 ~ 1.02)					

OR, Odds Ratio, CI, Confidence Interval.

Differences were considered statistically significant at P<0.05. Bold values indicate values with P < 0.05.

**Figure 3:**
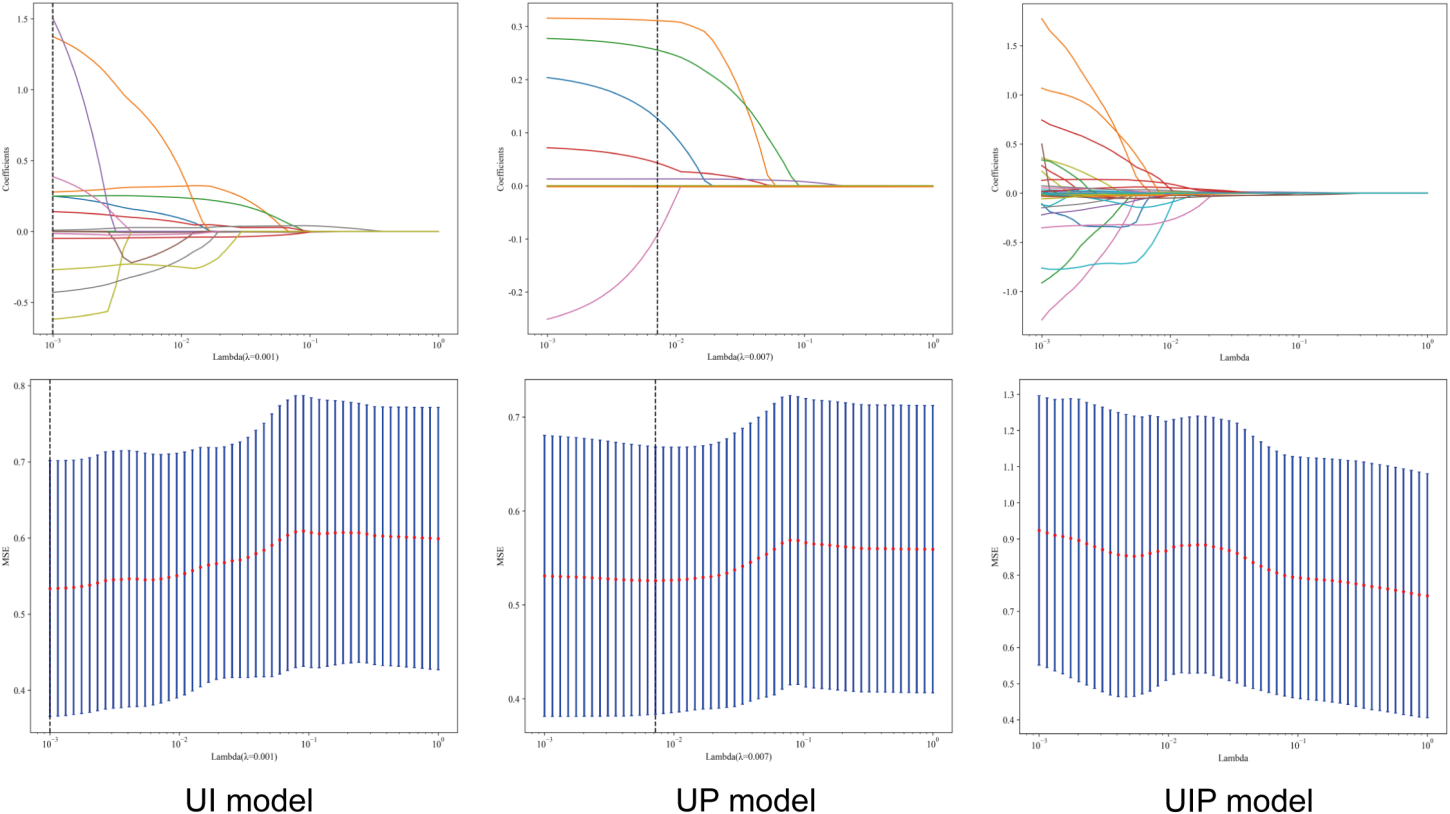
Lasso feature selection; Coefficients and MSE of 5 fold cross validation.

**Figure 4:**
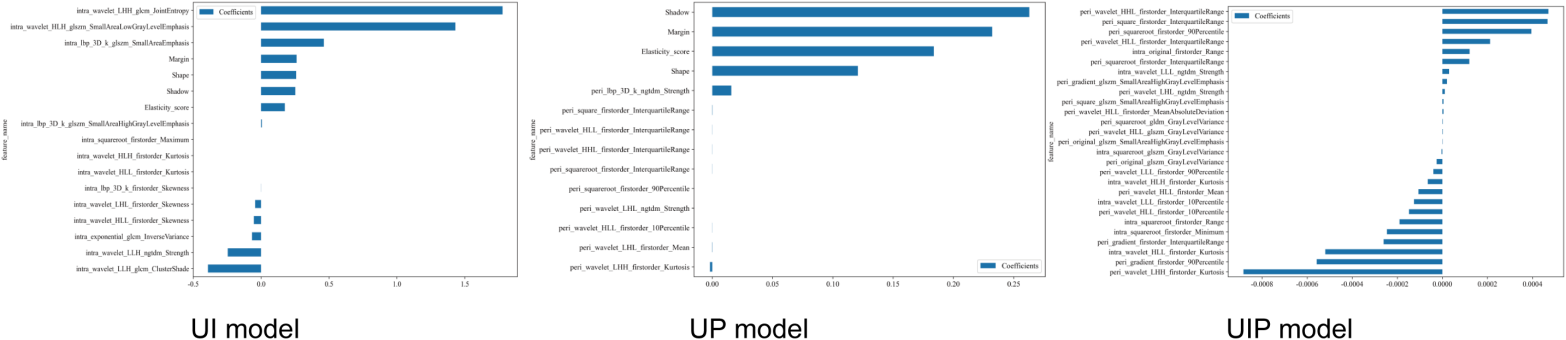
The histogram of the each models based on the selected features.

1561 radiomic features were extracted for the tumor interior and the tumor periphery, including 14 shape-based features; 306 first-order statistics; and 1241 second-order features. The extracted features were screened out based on the independent sample t-test (P < 0.05) for 129,57and 198 intratumoral, peritumoral and intratumoral-peritumoral dual-region features respectively. Then, 35,24 and 59 selected features were filtered based on the correlation coefficient. Finally, using the LASSO regression algorithm, the non-zero coefficients of the features were reduced to 14,15 and 24 predictive features respectively. Coefficients and mean standard error of 5 folds validation is show in **Figure 3**. Rad score is show as follow, **Figure 4** shows the coefficients value in the final selected non-zero features. Then these features were input into machine learning models to construct ultrasound imaging radiomics models for intratumoral, peritumoral and intratumoral-peritumoral dual-region.

### 3.1 Clinical-ultrasound features model

**Figure 5:**
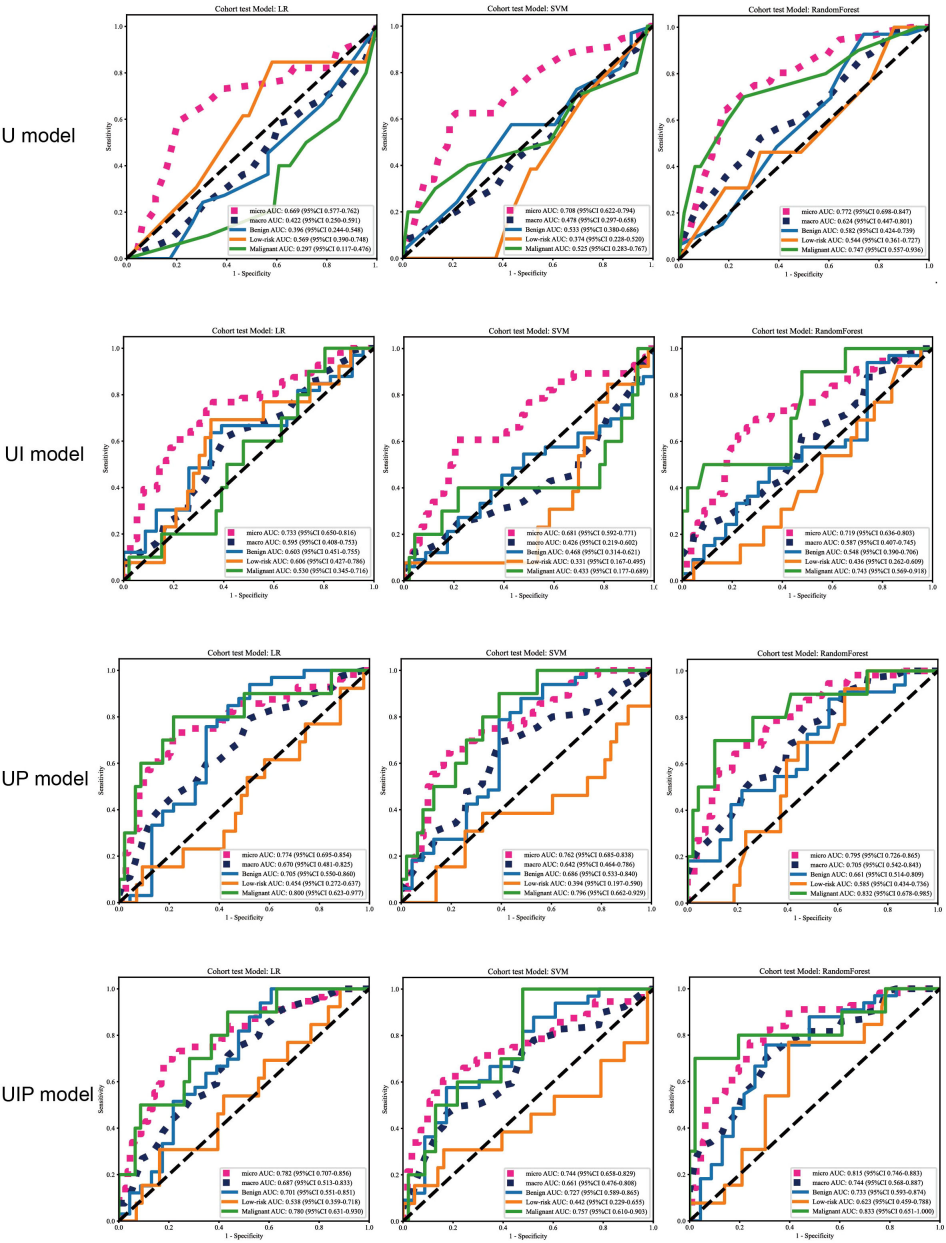
The ROC curves of different models in the test cohort.

As shown in **Table 3** & **Table 4**, the RF model achieved the highest accuracy (0.647 train,0.643 test)and micro-AUC(0.805 train,0.772 test),and also demonstrates the most stable ROC performance across different nodule classifications (**Figure 5**). SVM model showed moderate performance, with a slight decrease in test performance (accuracy: 0.593 train vs. 0.571 test),which was the worst performance among the three models. The accuracy of the LR model was moderate, but its micro-AUC was the lowest among the three models (0.661train,0.669test).

**Table 3:** The accuracy for each tumor type.

Cohort	Model	Accuracy	Cohort	Model	Accuracy
Train	U-LR	0.588	Test	U-LR	0.589
	U-SVM	0.593		U-SVM	0.571
	U-RF	0.647		U-RF	0.643
	UI-LR	0.588		UI-LR	0.589
	UI-SVM	0.588		UI-SVM	0.589
	UI-RF	1		UI-RF	0.589
	UP-LR	0.62		UP-LR	0.643
	UP-SVM	0.606		UP-SVM	0.625
	UP-RF	1		UP-RF	0.625
	UIP-LR	0.615		UIP-LR	0.643
	UIP-SVM	0.606		UIP-SVM	0.625
	UIP-RF	1		UIP-RF	0.661

**Table 4:** Results of clinical-ultrasound, intratumoral and peritumoral radiomics features models.

Group	Cohort	Model	Label	Acc	AUC	95% CI	Sensitivity	Specificity	PPV	NPV	Precision	Recall	F1	Threshold
U-model	Train	LR	Micro-AUC	0.724	0.661	0.614-0.707	0.584	0.794	0.586	0.792	0.586	0.584	0.585	0.451
			Benign	0.588	0.379	0.304-0.454	1	0	0.588	0	0.588	1	0.741	0.451
			Low-risk	0.774	0.489	0.400-0.578	0	1	0	0.774	0	0	0	0.246
			Malignant	0.814	0.363	0.270-0.457	0	1	0	0.814	0	0	0	0.19
		SVM	Micro-AUC	0.732	0.712	0.669-0.756	0.561	0.817	0.605	0.788	0.605	0.561	0.582	0.417
			Benign	0.615	0.531	0.455-0.608	0.992	0.077	0.606	0.875	0.606	0.992	0.752	0.606
			Low-risk	0.774	0.497	0.410-0.585	0	1	0	0.774	0	0	0	0.226
			Malignant	0.842	0.488	0.376-0.601	0.171	0.994	0.875	0.84	0.875	0.171	0.286	0.417
		RF	Micro-AUC	0.771	0.805	0.770-0.840	0.584	0.864	0.683	0.806	0.683	0.584	0.629	0.34
			Benign	0.661	0.69	0.620-0.760	0.977	0.209	0.638	0.864	0.638	0.977	0.772	0.606
			Low-risk	0.783	0.639	0.555-0.723	0.04	1	1	0.781	1	0.04	0.077	0.235
			Malignant	0.851	0.779	0.697-0.861	0.341	0.967	0.7	0.866	0.7	0.341	0.459	0.267
	Test	LR	Micro-AUC	0.726	0.669	0.577-0.762	0.589	0.795	0.589	0.795	0.589	0.589	0.589	0.516
			Benign	0.589	0.396	0.244-0.548	1	0	0.589	0	0.589	1	0.742	0.516
			Low-risk	0.768	0.569	0.390-0.748	0	1	0	0.768	0	0	0	0.211
			Malignant	0.821	0.297	0.117-0.476	0	1	0	0.821	0	0	0	0.19
		SVM	Micro-AUC	0.72	0.708	0.622-0.794	0.536	0.812	0.588	0.778	0.588	0.536	0.561	0.417
			Benign	0.607	0.533	0.380-0.686	0.97	0.087	0.604	0.667	0.604	0.97	0.744	0.606
			Low-risk	0.768	0.374	0.228-0.520	0	1	0	0.768	0	0	0	0.225
			Malignant	0.839	0.525	0.283-0.767	0.2	0.978	0.667	0.849	0.667	0.2	0.308	0.417
		RF	Micro-AUC	0.732	0.772	0.698-0.847	0.5	0.848	0.622	0.772	0.622	0.5	0.554	0.43
			Benign	0.679	0.582	0.424-0.739	0.97	0.261	0.653	0.857	0.653	0.97	0.78	0.43
			Low-risk	0.768	0.544	0.361-0.727	0	1	0	0.768	0	0	0	0.171
			Malignant	0.839	0.747	0.557-0.936	0.4	0.935	0.571	0.878	0.571	0.4	0.471	0.267
UI-model	Train	LR	Micro-AUC	0.706	0.711	0.668-0.753	0.357	0.88	0.598	0.733	0.598	0.357	0.448	0.298
			Benign	0.588	0.543	0.466-0.619	1	0	0.588	0	0.588	1	0.741	0.662
			Low-risk	0.774	0.54	0.454-0.627	0	1	0	0.774	0	0	0	0.196
			Malignant	0.814	0.528	0.439-0.616	0	1	0	0.814	0	0	0	0.17
		SVM	Micro-AUC	0.725	0.717	0.676-0.759	0.588	0.794	0.588	0.794	0.588	0.588	0.588	0.234
			Benign	0.588	0.52	0.443-0.598	1	0	0.588	0	0.588	1	0.741	0.589
			Low-risk	0.774	0.55	0.459-0.642	0	1	0	0.774	0	0	0	0.229
			Malignant	0.814	0.622	0.536-0.707	0	1	0	0.814	0	0	0	0.186
		RF	Micro-AUC	1	1	1.000-1.000	1	1	1	1	1	1	1	0.515
			Benign	1	1	1.000-1.000	1	1	1	1	1	1	1	0.698
			Low-risk	1	1	1.000-1.000	1	1	1	1	1	1	1	0.515
			Malignant	1	1	1.000-1.000	1	1	1	1	1	1	1	0.538
	Test	LR	Micro-AUC	0.744	0.733	0.650-0.816	0.375	0.929	0.724	0.748	0.724	0.375	0.494	0.266
			Benign	0.589	0.603	0.451-0.755	1	0	0.589	0	0.589	1	0.742	0.5
			Low-risk	0.768	0.606	0.427-0.786	0	1	0	0.768	0	0	0	0.266
			Malignant	0.821	0.53	0.345-0.716	0	1	0	0.821	0	0	0	0.188
		SVM	Micro-AUC	0.726	0.681	0.592-0.771	0.589	0.795	0.589	0.795	0.589	0.589	0.589	0.235
			Benign	0.589	0.468	0.314-0.621	1	0	0.589	0	0.589	1	0.742	0.586
			Low-risk	0.768	0.331	0.167-0.495	0	1	0	0.768	0	0	0	0.235
			Malignant	0.821	0.433	0.177-0.689	0	1	0	0.821	0	0	0	0.188
		RF	Micro-AUC	0.708	0.719	0.636-0.803	0.464	0.83	0.578	0.756	0.578	0.464	0.515	0.36
			Benign	0.625	0.548	0.390-0.706	0.909	0.217	0.625	0.625	0.625	0.909	0.741	0.418
			Low-risk	0.696	0.436	0.262-0.609	0.077	0.884	0.167	0.76	0.167	0.077	0.105	0.098
			Malignant	0.857	0.743	0.569-0.918	0.2	1	1	0.852	1	0.2	0.333	0.142
UP-model	Train	LR	Micro-AUC	0.744	0.778	0.740-0.815	0.475	0.878	0.66	0.77	0.66	0.475	0.553	0.372
			Benign	0.652	0.663	0.591-0.736	0.938	0.242	0.639	0.733	0.639	0.938	0.76	0.62
			Low-risk	0.778	0.633	0.550-0.717	0.12	0.971	0.545	0.79	0.545	0.12	0.197	0.213
			Malignant	0.81	0.71	0.620-0.800	0.22	0.944	0.474	0.842	0.474	0.22	0.3	0.229
		SVM	Micro-AUC	0.735	0.751	0.711-0.790	0.593	0.805	0.604	0.798	0.604	0.593	0.598	0.321
			Benign	0.62	0.647	0.572-0.721	0.985	0.099	0.61	0.818	0.61	0.985	0.753	0.6
			Low-risk	0.774	0.541	0.449-0.633	0	1	0	0.774	0	0	0	0.232
			Malignant	0.819	0.733	0.649-0.817	0.146	0.972	0.545	0.833	0.545	0.146	0.231	0.169
		RF	Micro-AUC	1	1	1.000-1.000	1	1	1	1	1	1	1	0.529
			Benign	1	1	1.000-1.000	1	1	1	1	1	1	1	0.674
			Low-risk	1	1	1.000-1.000	1	1	1	1	1	1	1	0.543
			Malignant	1	1	1.000-1.000	1	1	1	1	1	1	1	0.529
	Test	LR	Micro-AUC	0.774	0.774	0.695-0.854	0.482	0.92	0.75	0.78	0.75	0.482	0.587	0.35
			Benign	0.714	0.705	0.550-0.860	1	0.304	0.673	1	0.673	1	0.805	0.434
			Low-risk	0.732	0.454	0.272-0.637	0	0.953	0	0.759	0	0	0	0.353
			Malignant	0.839	0.8	0.623-0.977	0.3	0.957	0.6	0.863	0.6	0.3	0.4	0.252
		SVM	Micro-AUC	0.756	0.762	0.685-0.838	0.607	0.83	0.642	0.809	0.642	0.607	0.624	0.443
			Benign	0.696	0.686	0.533-0.840	1	0.261	0.66	1	0.66	1	0.795	0.595
			Low-risk	0.768	0.394	0.197-0.590	0	1	0	0.768	0	0	0	0.236
			Malignant	0.821	0.796	0.662-0.929	0.3	0.935	0.5	0.86	0.5	0.3	0.375	0.163
		RF	Micro-AUC	0.774	0.795	0.726-0.865	0.554	0.884	0.705	0.798	0.705	0.554	0.62	0.387
			Benign	0.625	0.661	0.514-0.809	0.939	0.174	0.62	0.667	0.62	0.939	0.747	0.52
			Low-risk	0.768	0.585	0.434-0.736	0	1	0	0.768	0	0	0	0.173
			Malignant	0.857	0.832	0.678-0.985	0.4	0.957	0.667	0.88	0.667	0.4	0.5	0.284
UIP-model	Train	LR	Micro-AUC	0.754	0.784	0.748-0.821	0.48	0.891	0.688	0.774	0.688	0.48	0.565	0.34
			Benign	0.647	0.677	0.605-0.748	0.938	0.231	0.635	0.724	0.635	0.938	0.758	0.505
			Low-risk	0.783	0.659	0.577-0.742	0.12	0.977	0.6	0.791	0.6	0.12	0.2	0.205
			Malignant	0.801	0.72	0.632-0.807	0.195	0.939	0.421	0.837	0.421	0.195	0.267	0.183
		SVM	Micro-AUC	0.739	0.748	0.708-0.788	0.593	0.812	0.612	0.8	0.612	0.593	0.602	0.289
			Benign	0.611	0.637	0.563-0.711	0.992	0.066	0.603	0.857	0.603	0.992	0.75	0.596
			Low-risk	0.774	0.533	0.440-0.626	0	1	0	0.774	0	0	0	0.234
			Malignant	0.819	0.727	0.645-0.809	0.098	0.983	0.571	0.827	0.571	0.098	0.167	0.17
		RF	Micro-AUC	1	1	1.000-1.000	1	1	1	1	1	1	1	0.535
			Benign	1	1	1.000-1.000	1	1	1	1	1	1	1	0.632
			Low-risk	1	1	1.000-1.000	1	1	1	1	1	1	1	0.573
			Malignant	1	1	1.000-1.000	1	1	1	1	1	1	1	0.535
	Test	LR	Micro-AUC	0.732	0.782	0.707-0.856	0.429	0.884	0.649	0.756	0.649	0.429	0.516	0.407
			Benign	0.714	0.701	0.551-0.851	0.97	0.348	0.681	0.889	0.681	0.97	0.8	0.416
			Low-risk	0.75	0.538	0.359-0.718	0	0.977	0	0.764	0	0	0	0.322
			Malignant	0.821	0.78	0.631-0.930	0.4	0.913	0.5	0.875	0.5	0.4	0.444	0.202
		SVM	Micro-AUC	0.762	0.744	0.658-0.829	0.625	0.83	0.648	0.816	0.648	0.625	0.636	0.505
			Benign	0.679	0.727	0.589-0.865	1	0.217	0.647	1	0.647	1	0.786	0.599
			Low-risk	0.768	0.442	0.229-0.655	0	1	0	0.768	0	0	0	0.239
			Malignant	0.804	0.757	0.610-0.903	0.2	0.935	0.4	0.843	0.4	0.2	0.267	0.166
		RF	Micro-AUC	0.774	0.815	0.746-0.883	0.464	0.929	0.765	0.776	0.765	0.464	0.578	0.336
			Benign	0.661	0.733	0.593-0.874	0.879	0.348	0.659	0.667	0.659	0.879	0.753	0.48
			Low-risk	0.732	0.623	0.459-0.788	0.154	0.907	0.333	0.78	0.333	0.154	0.211	0.262
			Malignant	0.929	0.833	0.651-1.000	0.6	1	1	0.92	1	0.6	0.75	0.336

All models showed high sensitivity(>0.9) but low specificity(<0.3) for benign nodules, and high specificity(>0.9) but low sensitivity(<0.4) for malignant nodules, while performance was poor in low-risk group with near-zero sensitivity. Among them, RF achieved the highest F1-scores for malignant nodules (0.459 train, 0.471 test).

### 3.2 Ultrasound radiomics-based models

#### 3.2.1 Clinical-ultrasound and intratumoral radiomics features model

As shown in **Table 3** & **Table 4**, the RF model achieved a perfect score on all metrics (Accuracy=1.0, AUC = 1.0, Sensitivity=1.0, Specificity=1.0)in training cohorts but a significant performance drop on the test cohort, confirming the overfitting of the data.

The accuracy of the LR model in the UI model was consistent with that in the U model, and also with that of the SVM model in the UI model(0.589 test).However, LR’s test micro-AUC was higher than SVM model(0.733 vs 0.681)).The ROC of LR model was also higher than that of SVM model in different nodule classifications (**Figure 5**).

All models were highly biased in assessing benign, low-risk, and malignant nodules, with sensitivity and specificity data close to 0 or 1.

#### 3.2.2 Clinical-ultrasound and peritumoral radiomics features model

As shown in **Tables 3**, **4**, the RF model showed a clear overfitting performance again.

LR model showed slightly higher accuracy than SVM model(0.643 test vs 0.625 test), and both higher than that in UI model. The same is true in terms of ROC (**Figure 5**).

Notably, in the malignant nodule group, although the sensitivity(=0.3) was still low, the AUC and F1 of the models were significantly increased compared with U models in test cohort(UP model(LR AUC 0.8,F1 0.4;SVM AUC 0.796,F1 0.375) vs U model(LR AUC 0.297,F1 0.0;SVM AUC 0.525,F1 0.0) vs UI model(LR AUC 0.53,F1 0.0;SVM AUC 0.433,F1 0.0)).

#### 3.2.3 Clinical-ultrasound and intratumoral-peritumoral dual-region radiomics features model

The accuracy of LR model and SVM model in UIP model was consistent with that in UP model(test 0.643,0.625), and the accuracy and ROC of LR model was higher than that of SVM model.

Despite its overfitting, the RF model’s test performance for the malignant group was the highest of all models(accuracy 0.929, AUC 0.833,sensitivity 0.60,NPV 0.92,F1 0.75), as shown in **Tables 3**, **4**, **Figure 5**.

**Figure 6:**
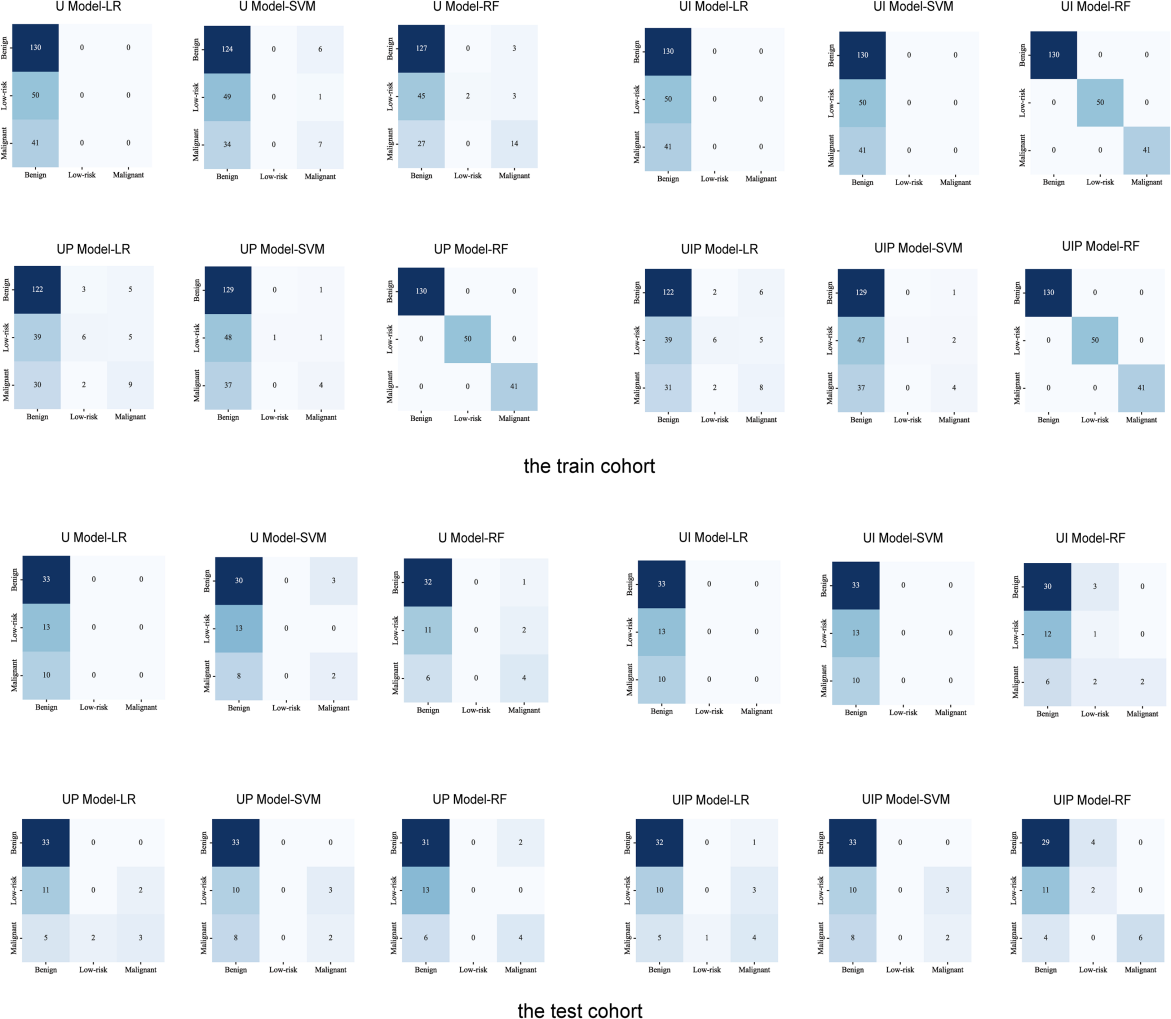
Confusion matrices showing classification performance for each tumor type. abscissa:actual class; ordinate:predicated class The numbers in the cells represent the number of patients predicted by the model.

**Figure 6** presents the confusion matrices for the all models, showing the classification performance for the three tumor classifications.

### 3.3 Comparison of diagnostic efficacy between UP-LR model and TI-RADS

ACR-TIRADS was used to classify all included nodules as benign, low-risk, and malignant. We performed ROC curve analysis in three separate rounds(benign vs low-risk and malignant; low-risk vs benign and malignant; malignant vs benign and low-risk). In each round, the target TI-RADS category and its corresponding pathological result were coded as 0, while all other categories and pathological types were combined and coded as 1. ROC curve analysis was then employed to calculate the AUC and its confidence interval for that specific category and then compared with that of the best-performing model (UP-LR model),as showed in **Table 5**. In the diagnosis of benign nodules, the AUC of the UP-LR model was 0.705(95% CI 0.550–0.860); the AUC of TI-RADS was 0.525 (95% CI 0.525–0.659); In the diagnosis of low-risk nodules, the AUC of the UP-LR model was 0.454 (95% CI 0.272–0.637); the AUC of TI-RADS was 0.461 (95% CI 0.38–0.543);In the diagnosis of malignant nodules, the AUC of the UP-LR model was 0.8 (95% CI 0.623–0.977); the AUC of TI-RADS was 0.525 (95% CI 0.435–0.615).

**Table 5:** Comparison of ROC analysis between UP-LR model and TI-RADS.

Area under the curve				
Group	Label	Area	Asymptotic 95% confidence interval	
			Lower bound	Upper bound
TI-RADS	begnin	0.592	0.525	0.659
	low-risk	0.461	0.38	0.543
	malignent	0.525	0.435	0.615
UP model	Benign	0.705	0.55	0.86
	Low-risk	0.454	0.272	0.637
	Malignant	0.8	0.623	0.977

a Under the nonparametric assumption.

b Null hypothesis: true area = 0.5.

### 3.4 SHapley Additive exPlanations of the best-performing model

**Figure 7:**
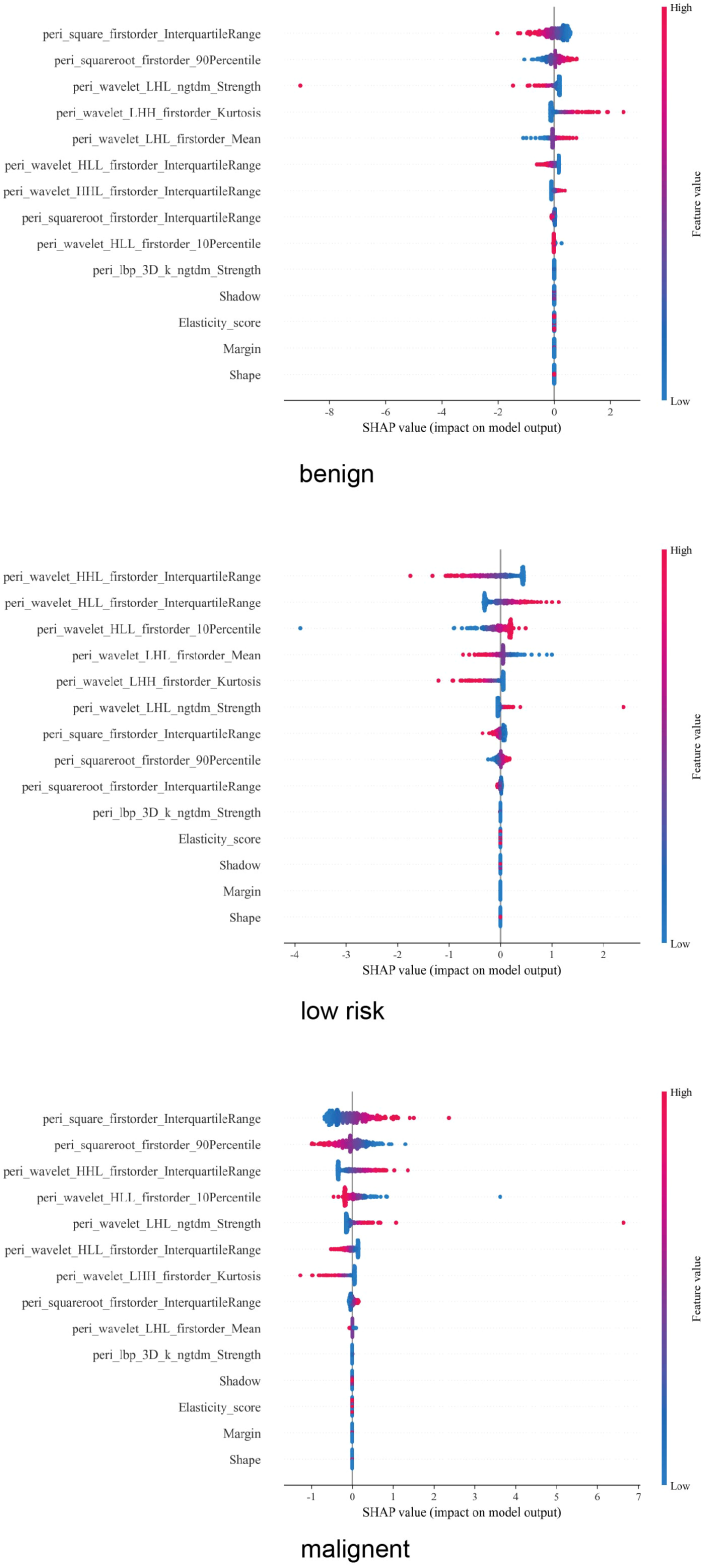
SHAP method of UP-LR model in diagnosing follicular nodules.

As shown in **Figure 7**, for the three-category classification of thyroid nodules, the top-ranked features are almost exclusively peritumoral radiomics features, particularly wavelet-transformed first-order statistics (such as interquartile range and percentiles) and texture features (e.g., ngtdm_Strength). Traditional ultrasound features generally rank lower than the radiomics features. This highlights the importance and necessity of incorporating peritumoral regions into the analysis. From benign to low-risk to malignant nodules, as the invasiveness of the nodules increases, the values of these features tend to be higher and are more frequently associated with positive SHAP values.

## 4 Discussion

Preoperative diagnosis of thyroid follicular tumors is a research hotspot and always a challenge in clinical practice. However, as we understand, there are few articles focusing on the differentiation of tumors classified as FA, FT-UMP, and FTC. The challenge lies in determining the presence of invasive growth, particularly capsular invasion and vascular invasion (16, 21). Most radiomics studies focus on the features within the tumor and ignore the key biological features available in the surrounding region (22, 23).Therefore, based on ultrasound images, we have developed intratumoral and peritumoral radiomic models to explore better methods for distinguishing tumor classifications. Combining intratumoral and peritumoral radiomics, which captures the interplay between tumor biology and the microenvironment, may surpass methods involving only intratumoral features and could lead to novel applications in thyroid tumor radiomics (24, 25)Three machine learning models, LR,SVM and RF, were used to establish U,UI,UP and UIP models respectively by using clinical and ultrasound features, intratumoral radiomics and peritumoral radiomics. From the results, it can be seen that model LR in model UIP has better stability and generalization ability.

While the overall accuracy is low, the UP-LR model shows promising discrimination among benign, low-risk, and malignant cases(all AUC >0.70),with the highest performance observed in the malignant nodules.

Compared with the commonly used TI-RADS model, the diagnostic efficiency of this model for benign and malignant nodules is significantly higher. In diagnosing benign nodules, the model achieves a sensitivity of 1.00, ensuring that all benign nodules were identified, but its low specificity led to the possibility of misdiagnosis. This means that if the model classifies a nodule as non-benign, the result is highly reliable (NPV = 1.0). For diagnosing malignant and low-risk nodules, the model exhibited extremely high specificity(both >0.95), especially for malignant nodules (PPV = 0.6, specificity = 0.957),which means if the model classifies a nodule as malignant or low-risk, the result is highly reliable and further diagnostic confirmation is recommended. Notably, the AUC was 0.8 for malignant nodules and only 0.454 for low-risk nodules. It shows that the model performed poorly in identifying low-risk nodules. This may be related to the insufficient number of low-risk samples in the dataset and the significant overlap of features between low-risk and benign and malignant nodules. Indeed, incorporating the more challenging FT-UMP categories into the three-classification problem may indeed lead to a decrease in the overall performance of the model (26). FT-UMP may partially overlap with FTA and FTC in imaging, increasing the difficulty of classification. FT-UMP is diagnostically challenging, even for pathologists, which is also reflected in our models. Specifically, we suggest that this model can be used as a complementary tool after inconclusive FNA results (Bethesda III/IV) to help prioritize high-risk patients for surgery and reduce unnecessary interventions in low-risk cases.

The RF model demonstrated the best performance in the U model, with high accuracy and AUC. However, in the UI, UP, and UIP models, it exhibited significant overfitting. Although this model showed the highest diagnostic value for malignant nodules (AUC 0.833), this resulted in considerable instability when applied to unknown data. Therefore, it is not recommended as a primary screening tool but could be considered as a secondary confirmation tool for high-risk nodules. In contrast, LR and SVM models exhibited better stability, though SVM’s overall efficacy was lower than that of LR. Considering the performance of all models, the LR model achieved higher AUC and accuracy in the test cohort, particularly in the UP model, which outperformed the UIP model in accuracy, specificity, PPV, sensitivity, and F1 score. We observed that incorporating intratumoral radiomic features did not improve model performance; instead, it led to a decline in certain key metrics. This suggests that intratumoral radiomic features may be redundant with peritumoral or clinical and ultrasound features, indicating that peritumoral radiomic features provide more critical discriminative information, which is similar with some previous studies (24, 27).

As previously described, follicular tumors share a similar underlying structure. Histologically, they form follicular structures resembling the normal thyroid gland, albeit with varying degrees of differentiation and spatial arrangement. The key diagnostic criteria revolve around the presence of invasive growth—specifically, its type and extent. A diagnosis of FTC requires evidence of definite vascular invasion or extensive capsular invasion. In contrast, FT-UMP represents a borderline category where the pathologist observes suspicious or focal signs of invasion under the microscope, which are insufficient to meet the strict diagnostic thresholds for FTC (28). This pathological continuum explains the considerable challenge in distinguishing these entities in our radiomics study, as their essential difference lies in microscopic invasive behavior rather than macroscopic imaging findings. We aimed to capture more discriminative information regarding this behavior through the selection of peritumoral radiomics features.

When capsular or vascular invasion occurs in a thyroid nodule, remodeling of the extracellular matrix, increased local echogenicity heterogeneity, and ill-defined margins may contribute to elevated tissue heterogeneity in the peritumoral region. These changes are reflected in radiomic features such as InterquartileRange and Variance. The formation of neovascularization and tumor thrombi may correlate with high-intensity image traits, including the 90th Percentile, Maximum, and various HighGrayLevelEmphasis features. Meanwhile, attributes such as Strength may also indicate underlying tissue destruction and remodeling processes (5, 29).

These observations align with the results of our SHAP analysis. In the three-class classification of thyroid nodules, all peritumoral radiomics features were ranked as top contributors, outperforming traditional ultrasound characteristics. This reinforces the proposition that peritumoral radiomics features serve as the most powerful indicators in our model for stratifying tumor risk, capturing information beyond conventional visual assessment criteria. It further validates the value and necessity of our study design incorporating the peritumoral region. With increasing nodule aggressiveness, the values of these imaging features tend to rise and more frequently assume positive values—a pattern fully consistent with clinical understanding and supportive of the rationality of the model’s decision logic. Thereby, the model’s applicability and reliability in clinical practice are considerably enhanced.

Our study also has certain limitations. First, we agree that the poorer performance of the low-risk category (FT-UMP) is a major limitation. We will recommend that future studies integrate molecular markers, such as mutational analysis, with radiomics to improve the classification of critical categories such as FT-UMP. Second, the sample size of our study was limited and all were from a single center, which showed overfitting results. Therefore, we need to expand the sample size or conduct multicenter studies for external validation to further refine our models. In this study, only gray-scale ultrasound images were used to extract radiomics features. We found that the existing multimodal ultrasound images (contrast-enhanced ultrasound, elastic ultrasound) were also used to obtain radiomics features to understand the lesions more comprehensively (30–32). To address this issue, further prospective studies could be conducted to improve the prediction accuracy in our future work.

## 5 Conclusion

The logistic regression model incorporating clinical-ultrasound features along with peritumoral radiomic features achieved stable performance in distinguishing between benign and malignant follicular thyroid tumors. It offers a valuable reference for clinical decision-making in the management of follicular thyroid tumors.

## Data Availability

The original contributions presented in the study are included in the article/supplementary material. Further inquiries can be directed to the corresponding author.
